# The hallmarks of CMV-specific CD8 T-cell differentiation

**DOI:** 10.1007/s00430-019-00608-7

**Published:** 2019-04-13

**Authors:** Sara P. H. van den Berg, Iris N. Pardieck, Josien Lanfermeijer, Delphine Sauce, Paul Klenerman, Debbie van Baarle, Ramon Arens

**Affiliations:** 1 Center for Infectious Disease Control, National Institute for Public Health and the Environment, Bilthoven, The Netherlands; 2 Laboratory of Translational Immunology, Department of Immunology, University Medical Center Utrecht, Utrecht University, Utrecht, The Netherlands; 3 Department of Immunohematology and Blood Transfusion, Leiden University Medical Center, Albinusdreef 2, 2333 ZA Leiden, The Netherlands; 4 Sorbonne Université, INSERM, Centre d’Immunologie et des Maladies Infectieuses (CIMI-Paris), Paris, France; 5 Nuffield Department of Medicine, Peter Medawar Building for Pathogen Research, University of Oxford, Oxford, UK; 6 NIHR Biomedical Research Centre, John Radcliffe Hospital, Oxford, UK

**Keywords:** Cytomegalovirus, CD8 T cell, Differentiation, Phenotype

## Abstract

Upon cytomegalovirus (CMV) infection, large T-cell responses are elicited that remain high or even increase over time, a phenomenon named memory T-cell inflation. Besides, the maintained robust T-cell response, CMV-specific T cells seem to have a distinctive phenotype, characterized by an advanced differentiation state. Here, we will review this “special” differentiation status by discussing the cellular phenotype based on the expression of CD45 isoforms, costimulatory, inhibitory and natural killer receptors, adhesion and lymphocyte homing molecules, transcription factors, cytokines and cytotoxic molecules. In addition, we focus on whether the differentiation state of CMV-specific CD8 T cells is unique in comparison with other chronic viruses and we will discuss the possible impact of factors such as antigen exposure and aging on the advanced differentiation status of CMV-specific CD8 T cells.

## Introduction

Human cytomegalovirus (HCMV), a beta-herpesvirus family member, infects around 60% of the worldwide population [1]. In healthy individuals, HCMV establishes a persistent latent infection with episodes of reactivation. Although HCMV infection is usually asymptomatic, in immunocompromised (e.g., HCMV-seronegative recipients receiving organs of HCMV-positive donors) and immune immature individuals (neonates), HCMV can cause serious disease [2].

A remarkable feature of HCMV infection is the capacity to elicit large T-cell responses that do not follow the typical contraction pattern after primary infection. Instead, the percentages of CMV-specific T cells remain high or even increase over time [3], a phenomenon named memory T-cell inflation [4, 5]. In the Western world, frequencies around 10% of HCMV-specific T cells of the total memory T-cell pool are commonly observed (with outliers > 50%), and this is found in both healthy and immunocompromised individuals [6, 7]. In elderly, the frequency of circulating HCMV-specific T cells is higher than in younger adults, and the reactivity of these cells can be restricted to a limited number of epitopes [8–11]. The increase in frequency of HCMV-specific CD8 T cells with age is also observed in studies with immunocompromised individuals and is similar to frequencies found in healthy donors [12].

Besides the sustained large T-cell response, the phenotype of CMV-specific T cells seems to be characteristic as well, typified by an advanced differentiation state. Here, we discuss the particulars of this “special” differentiation phenotype and asked the question whether the differentiation state of CMV-specific CD8 T cells is unique. In addition, we discuss the potential impact of antigen exposure and aging on the differentiation status of CMV-specific CD8 T cells.

## The differentiation phenotype of CMV-specific CD8 T cells

### CD45 isoforms

Isoforms of the protein tyrosine phosphatase CD45 are expressed at various levels on hematopoietic cell lineages. The high-molecular-weight isoform CD45RA is expressed by naïve T cells, while the low molecular weight isoform CD45RO is expressed on activated and memory T cells and is implicated in increasing the sensitivity of TCR signalling [13]. Advanced differentiation of T cells is, however, characterized by a lack of CD45RO while CD45RA is re-expressed. A large proportion of the HCMV-specific T cells have the latter phenotype (in combination with downregulation of costimulatory molecules, this phenotype is also called TEMRA), and this seems quite unique for HCMV [14]. For example, Epstein–Barr virus (EBV)-specific CD8 T cells are predominantly CD45RO positive [15] and human immunodeficiency virus (HIV)-specific T cells express lower levels of CD45RA [16].

### Costimulatory and inhibitory receptors

The advanced differentiation state of CMV-specific T cells is also marked by the lack of expression of the costimulatory receptors CD27 and CD28, which are otherwise constitutively expressed on naïve T cells [17]. This is in contrast to other virus-specific CD8 T cells. For example, EBV and hepatitis C virus (HCV)-specific T cells more often display expression of CD27 and CD28, and HIV-specific CD8 T cells, despite advanced loss of CD28, still express CD27 [17], although this may also depend on the disease state [18].

Acute HCMV infections frequently occur in CMV-negative transplant recipients receiving a CMV-positive organ. In these individuals, the CMV-specific T-cell response consists of mainly CD27^+^CD28^−^CD45RA^−^CD45RO^+^ memory T cells shortly after the peak of CMV infection [19]. In time, expression of CD27 is lost and CD45RA is re-expressed on the majority of the cells [20, 21]. The gradual loss of CD27 is also observed in mouse models, and is likely caused by chronic antigenic triggering [22].

In mouse models, the functional role of CD27 and CD28 has been studied in CMV infection and indicated that CD28 costimulation is especially important during primary infection to enhance CMV-specific T-cell expansion while CD27 and its ligand CD70 seem to play an activating role during both the primary and latent phase of infection [22–26]. The costimulatory receptor OX40 is transiently upregulated upon activation, and is important during the latent phase [27].

Programmed cell death 1 (PD-1), cytotoxic T-lymphocyte antigen 4 (CTLA-4), T-cell immunoglobulin domain and mucin domain protein 3 (TIM-3), lymphocyte activation gene 3 (LAG-3) and CD160 are inhibitory receptors associated with the exhaustion phenotype of T cells [28]. PD-1 was identified to be abundant on chronic lymphocytic choriomeningitis virus (LCMV)-specific T cells in mice models [29] and was next shown to be upregulated on T cells in a number of chronic viral infections including HIV [30, 31], hepatitis B virus (HBV) [32] or HCV [33]. In addition, PD-1 and other inhibitory molecules are abundantly found on T cells in the tumor microenvironment and this aspect forms the basis for reinforcing exhausted T cells by blocking these inhibitory molecules [34]. Indeed, as demonstrated by variants of LCMV eliciting either acute or chronic infection, the induction of the exhausted phenotype is caused by strong chronic antigenic triggering [35], and is elevated by the lack of CD4 T-cell help [29, 36].

Interestingly, during the latent phase, circulating CMV-specific T cells express relatively low levels of inhibitory receptors [37, 38]. PD-1 expression on CMV-specific T cells is lower compared to chronic virus-specific T cells against HBV [32], HIV [30, 38–40] and EBV-specific T cells [37, 38]. Likewise, also TIM-3, CD160 and 2B4 are expressed at lower levels in CMV-specific T cells compared to HIV-specific T cells [40]. Nonetheless, the inter-individual variation of PD-1 and 2B4 expression observed for CMV-specific T cells can be substantial [33, 40]. This heterogeneity of PD-1 expression could reflect different differentiation phenotypes of virus-specific memory T cells [38], and this may be independent of their capacity to control viruses. Indeed, PD-1 expression is not associated with functional capacity (e.g., secretion of cytokines and degranulation). In addition, data showed that CD8 T cells can further up-regulate PD-1 when they are activated [38]. Altogether this suggests that PD-1, expressed on CMV-specific T cells, is independent of T-cell exhaustion.

### Natural killer receptors

Although originally reported as natural killer cell receptors (NKRs), a number of these receptors such as immunoglobulin-like receptors (KIRs, LIRs such as CD85j) and lectin-like receptors (CD94/NKG2, KLRG1) are also expressed on CD8 T cells [41]. These molecules are likely implicated in the fine-tuning of the anti-viral response. Indeed, primary CMV infection induces an increased expression of both inhibitory and activating NKRs, which remains high during the latent infection phase, while viral load is undetectable [42]. Yet, the precise role of different NKRs remains to be determined.

Similarity between CMV-specific T cells and other chronic viruses is found in the expression patterns for several inhibiting NKRs. CMV-specific T cells, just like EBV and HIV-specific T cells, show substantial expression of CD85j (ILT2/LIR-1) compared to the overall T-cell pool [43–45]. This CD85j expression is most abundant in TEMRAs and CD28^−^ CD8 T cells [44], suggesting an advanced differentiation phenotype. In addition, the overwhelming majority of CMV, EBV and HIV-specific T cells express KLRG1, often together with loss of expression of CD28 and CCR7, indicating that these cells have undergone multiple cell divisions but are still active in cytokine production [46, 47]. Also, expression of NKG2A is increased on CMV-specific CD8 T cells [42, 48]. However, KIRs do not seem upregulated on CMV-specific T cells and/or HIV-specific T cells: only small fractions express CD158 variants or NKB-1 (KIR3DL1) [43, 49]. Although increased expression of NKG2C on CD8 T cells is associated with CMV-seropositivity [42] and CMV-reactive T cells upon restimulation show NKG2C expression [44], CMV-specific T cells stained with MHC class I tetramers do not seem to express NKG2C [42, 50]. Overall, CD8 T cells specific for CMV show low expression of KIRs and NKG2C and increased expression of CD85j, NKG2A and KLRG1 during the latent phase of the infection.

CMV-specific CD8 T cells also express the NKRs CD56 and CD57. CD56^+^ CD8 T cells are known for their natural killer-like cytotoxicity [51], and CD56 is shown on CMV-specific T cells in renal transplant patients [52] and healthy individuals (unpublished observations, S. van den Berg and D. van Baarle). CD57 expression represents a cellular phenotype associated with poor proliferative capacity but high cytotoxic potential [53]. On CMV-specific T cells, CD57 expression, often co-expressed with CD85j, increases with age, but a large variation in expression exists [44, 54]. CD57 expression on CMV, EBV, and HIV-specific CD8 T cells was low to moderate in adults [32, 46], whereas others reported overall a high expression on these virus-specific T cells of CD57 in older subjects [55]. In the latter, CMV-specific T cells seem to express CD57 at higher levels than EBV and HIV-specific T cells, albeit not substantially.

### Adhesion molecules and lymphocyte homing

CMV-specific CD8 T cells are largely negative for CCR7 and CD62L [16, 17, 49], which are homing receptors for lymphoid organs. This property, which is shared with T cells specific for other chronic viruses, allows the cells to circulate throughout the body, and reside in peripheral tissue, spleen and blood.

CX3CR1, which recognizes fractalkine expressed by endothelial cells, is abundantly expressed by CMV-specific cells [10, 56] during the primary and latent infection, whereas CCR1 and CXCR6 are only present during the acute phase [37]. High and intermediate expression of CX3CR1 seems to be unique for CMV-specific CD8 T cells in both human and mice [37, 56], as the frequency of this chemokine receptor on EBV [57], HBV and HCV-specific T cells is much lower [58]. CMV-specific CD8 T cells with intermediate expression of CX3CR1 associate with self-renewal potential, but the role of CX3CR1 seems to be redundant, since memory T-cell inflation is unaltered in case of CX3CR1 deficiency [56]. In addition, CXCR3 is commonly expressed on CMV-specific T cells as well as EBV-specific T cells [57]. The homing cell adhesion molecule CD44 is uniformly high expressed on all CMV-specific T cells [48, 59].

### Transcription factors, cytokines and cytotoxic molecules

Transcription factors (TFs) are crucial regulators of cellular differentiation and function including the cytotoxic potential and cytokine secretion. For CD8 T cells, the TFs Eomes and T-bet are particularly useful to determine the functional profile. For example, T-bet^dim^ and Eomes^high^ expression profiles are associated with expression of exhaustion markers as observed in HIV-specific T cells, whereas many CMV and EBV-specific T cells exhibit intermediate levels of Eomes and high levels of T-bet [37, 40, 60]. Blimp-1 and the Homolog of Blimp-1 in T cells (Hobit) are also clearly expressed by CMV-specific CD8 T cells [61, 62].

Related to the above-described TF profile is the high granzyme B and perforin expression in CMV-specific CD8 T cells [39, 40, 49, 63]. These cells also abundantly produce IFN-γ and TNF after re-stimulation, while IL-2 is produced by only a subset of the inflationary CMV-specific CD8 T cells [63]. The expression of the above-described effector molecules is consistent with the functional non-exhaustion phenotype of CMV-specific T cells, and underlines their functional status and requirement for lifelong protection against viral dissemination [64]. Analysis of transcriptional networks in inflating cells reveals a module of genes strongly driven by T-bet, not seen in T-cell exhaustion [65].

The low IL-2 production may coincide with the reduced expression of IL-2Rβ (CD122/IL-15Rβ) on CMV-specific CD8 T cells [37, 48, 63, 66]. In addition, virus-specific effector CD8 T cells activated in vivo during primary EBV or CMV infection down-regulate IL-7Rα (CD127) and IL-15Rα (CD215) expression [67]. With time, CMV-specific CD8 T cells maintain high levels of IL-15Rα. This contrasts with the lower expression of IL7Rα on CMV-specific CD8 T cells compared to EBV-specific CD8 T cells [32, 68–70]. Interestingly, IL-7Rα expression was tightly associated with population size in blood [70]. However, this correlation was not sustained in tonsillar lymphoid tissue where CMV-specific T cells were less abundant than EBV-specific T cells, despite higher IL-7Rα expression [70].

## Is the advanced differentiated T-cell phenotype unique?

**Fig. 1:**
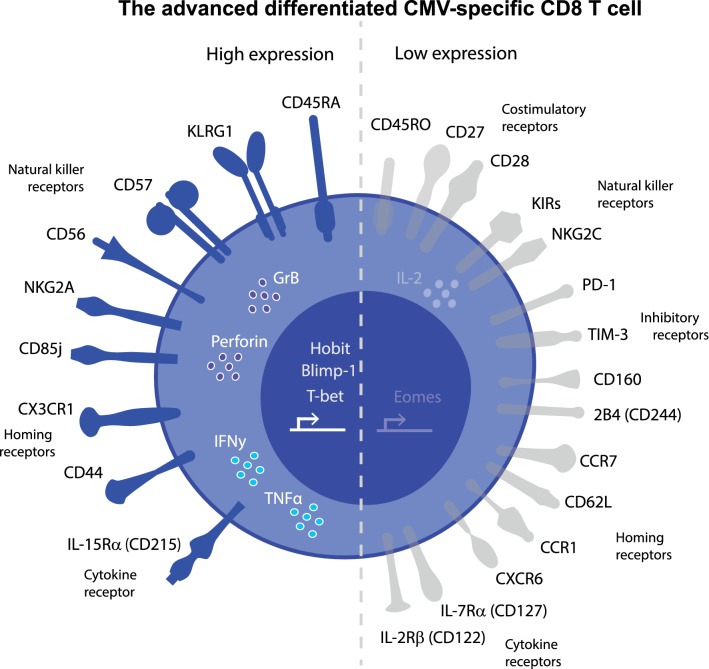
**The advanced differentiation phenotype of CMV-specific CD8 T cells**. The advanced differentiated CMV-specific CD8 T cells are typified by either expression or down-modulation of different surface receptors, cytokines and transcription factors. Surface receptors that are expressed are depicted in blue on the left side of the cell, whereas down-modulated or non-expressed surface receptors are depicted in gray on the right side of the cell. CMV-specific CD8 T cells express the CD45 isoform CD45RA, different natural killer receptors (CD85j, CD56, CD57, NKG2A and KLRG1), IL-15Rα and the homing receptors CX3CR1 and CD44. These cells do not express or lowly express CD45RO, costimulatory receptors CD27 and CD28, natural killer receptors (KIRs and NKG2C), inhibitory receptors (PD-1, TIM-3, CD160 and 2B4), homing receptors (CXCR6, CCR1, CD62L and CCR7) and cytokine receptors IL-2Rβ (CD122) and IL-7Rα (CD127). CMV-specific CD8 T cells have intermediate expression of the transcription factor Eomes and strong expression of transcription factors Hobit, Blimp-1 and T-bet. Related to this transcription profile is the high expression of cytokines IFN-γ and TNF-γ and the cytotoxic molecules granzyme B (GrB) and perforin. In general, IL-2 production by CMV-specific CD8 T cells is low

The above-described advanced differentiated CD8 T-cell phenotype is clearly observed for CMV-specific T cells, and could be considered as a distinct type of effector-memory (EM) T cells. The phenotype involves expression of inhibitory molecules such as KLRG1, CD57 and CD56, yet the cells are nevertheless functional with respect to cytokine production and cytotoxicity (Fig. 1). However, the advanced differentiated phenotype is not entirely exclusive as also other viruses can elicit CD8 T cells with a similar differentiation status. Less attention is given to this since either only a small subset among the total memory pool has this phenotype (e.g., upon infection with EBV or HIV) or the frequencies of the late-stage differentiated CD8 T cells are generally lower compared to those in CMV infection (e.g., infection with herpes simplex virus-1 (HSV-1) [71] and parvoviruses B19 and PARV4 [72]. One clear feature is that high doses of adenovirus-based vaccine vectors can actually induce a comparable phenotype (and transcriptome) to CMV [56], which is also accompanied with a high frequency of the cells, which makes this a vaccine platform with great potential. As variation exists in the differentiation state of CMV-specific T cells between individuals, we will next discuss factors that influence the T-cell differentiation.

## Establishment of the advanced differentiation phenotype

The phenotype of the CMV-specific CD8 T cells is strongly connected with the magnitude of the CMV-specific T-cell response. Cross-sectional human studies show that in both healthy and immunosuppressed individuals, a high HCMV-specific T-cell response is associated with a high percentage of advanced differentiated T cells within the total specific T-cell population [44, 73–75]. Nevertheless, the association between the differentiation state and level of CMV-specific T cells is shown in experimental mouse models [74, 76]. Low-dose inoculums elicit fewer circulating CMV-specific CD8 T cells, and these cells have a less advanced differentiation phenotype. Accordingly, interference with an established mouse CMV infection by antiviral treatment reduces the frequency of the CMV-specific CD8 T-cell response, and also in this setting, the CD8 T cells acquired a lesser differentiated phenotype compared to CMV-infected mice that are untreated [77].

Differences in the infectious dose of primary CMV infection may be instrumental in causing the large variation of the advanced-stage differentiation status of CMV-specific T cells that exists between individuals. CMV-specific CD8 T cells may reach an advanced differentiation phenotype already early after infection, and then maintain this status stably over time. In young individuals and even in children, advanced differentiated CMV-specific T cells can appear [78–80]. Thus, the (primary) infectious dose might determine the viral setpoint (the initial balance between virus and host after primary infection) [81] and thereby subsequently influence the level and amount of viral reactivation episodes and consequent antigen triggering of CMV-specific T cells.

Notably, within the inflationary epitope-specific memory T-cell population, not all CMV-specific T cells acquire the late-stage differentiation phenotype. Depending on the viral dose, a significant portion can attain a central-memory (CM) phenotype [76]. These CM-like CD8 T cells produce more IL-2 and are probably dominantly contributing to T-cell expansion upon re-challenge [82]. Also, within the total pool of CMV-specific T cells non-inflationary T cells exist directed against a distinct subset of epitopes, which never acquire the EM-like differentiation during the latent phase of infection [63]. In line with this are the observations that the enhanced differentiation state of the HCMV-specific T cell is observed for different epitopes [32]. A critical aspect for virus-specific T cells undergoing memory inflation or not, does not depend on the intrinsic property of the peptide epitope but on the context of viral gene expression. CMV epitopes that normally induce non-inflationary CD8 T-cell responses from its native site can induce an inflationary response due to C-terminal localization allowing better peptide processing, also leading to a more advanced differentiated phenotype [83, 84].

Besides the infectious dose, aging also impacts the differentiation status of the CMV-reactive T cells. In cross-sectional studies, it was observed that the number of HCMV-specific T cells increases over time [6, 47]. And this is accompanied by an increase of HCMV-specific cells that re-express CD45RA [11] and express KLRG1 [47]. Moreover, using new computational tools, it was recently shown that inflationary MCMV-specific T cells are progressively differentiating in time (based on the markers KLRG1, CD44, CD27 and CD62L), long after the initial infection [74, 85]. In line with these studies is the observation that telomeres of HCMV-specific CD8 T cells are significantly shorter compared to the corresponding phenotypic subsets of the total CD8 T-cell pool [86]. The shortest telomere lengths were found in old individuals compared to young individuals in all different memory subsets (based on CD27 and CD45RA distinction). Overall, this indicates that with aging CMV-specific cells undergo more proliferation and enhanced differentiation.

Important for the enhanced differentiation after CMV infection is the capacity of CMV to become latent. Essentially, latent genomes can sporadically desilence at certain genetic loci, which lead to gene expression of antigenic peptide-encoding genes without entering the productive cycle [87, 88]. This allows intermittent re-exposure of antigen to the virus-specific T cells, which keeps these cells “tickled” during a lifetime, but avoids continuous strong antigenic stimulation leading eventually to exhaustion as is the case for chronic infections with HIV or certain LCMV strains [89]. The large and gradual expansion of CMV-specific CD8 T cells with an enhanced differentiation phenotype could be interpreted as a lack of complete control of the virus. The T cells that show enhanced differentiation, thus, attempt to retain control over full reactivation of the virus. Accordingly, interference with an established MCMV infection by antiviral treatment reduces the frequency of the CMV-specific CD8 T-cell response, and also in this setting, the CD8 T cells reverted to a lesser differentiated phenotype compared to CMV-infected mice that were untreated [77]. It is generally assumed that the immune evasion strategies of CMV targeting the innate and adaptive immunity are critical for the long-term persistence of the virus [90, 91], but whether some of these strategies are capable of specifically modulating particular phenotypic characteristics of the CMV-specific T cell is unknown. The need and purpose of the maintenance of high-frequency CMV-specific CD8 T cells that progressively differentiate are, thus, unclear and may be driven by an ongoing shift in the virus–host equilibrium.

Another important aspect might be the broad tropism of CMV and its systemic spread as localized CMV infection results in less inflation and less advanced differentiation [92, 93]. The distinctive tropism of CMV, including the wide variety of target cells, innate immune cells such as myeloid cells as CMV vehicles, and the infrequent expression of immediate early genes leading to abortive reactivation, may thus, co-determine the fate of the T-cell response, and such characteristics may be the key differences compared to other chronic viruses that frequently reactivate, like EBV. Finally, the size of the genome of CMV is relatively large (compared to most other viruses), which may contribute to elicit larger T-cell responses and to the likelihood to encompass epitopes inducing inflationary T-cell responses.

## Concluding remarks

The characteristics of CMV-specific T cells, i.e., maintenance of high numbers and the late-differentiated EM-like phenotype, have been a subject of interest. Although the CMV-specific memory T-cell populations are diverse (in magnitude and phenotype) between individuals, it is evident that a large proportion of these cells are advanced differentiated. This particular phenotype seems to be related to the nature of CMV infection because it is more abundantly found upon CMV infection compared to other chronic viruses. The CMV-specific T cells are often late-stage differentiated T cells, have shorter telomeres and express inhibitory molecules such as KLRG1, CD57 and CD85j, yet the cells are nevertheless functional with respect to cytokine production and cytotoxicity [94]. Further studies are needed to unravel this seemingly conflicting feature of CMV-specific T cells. Large prospective studies in humans could provide further insight, but such studies may still be complicated given the possible impact of MHC heterogeneity in the human population compared to inbred mice [95]. Notably, the data discussed here reflect mainly the differentiation of the circulating CMV-specific T cells, which represents a subgroup of the total CD8 T-cell pool in the body. Whether a late-differentiated phenotype “uniquely” related to CMV infection is also present in the tissue-resident memory T-cell population remains to be elucidated. Several papers reveal a dual impact of CMV infection and aging on immune subsets [96–100]. Prevalence of CMV infection increases with age [101, 102], suggesting that CMV may take advantage over a senescent immune system. How long-term infection of CMV is able to change the virus–host balance leading to gradual higher levels of advanced differentiated T cells is unknown. Due to aging, immune control may gradually wane leading to more frequent reactivation.
